# The Tuberculosis Vaccine Candidate Bacillus Calmette-Guérin Δ*ureC*::*hly* Coexpressing Human Interleukin-7 or -18 Enhances Antigen-Specific T Cell Responses in Mice

**DOI:** 10.1371/journal.pone.0078966

**Published:** 2013-11-13

**Authors:** Martin Rao, Alexis Vogelzang, Peggy Kaiser, Stefanie Schuerer, Stefan H. E. Kaufmann, Martin Gengenbacher

**Affiliations:** Max Planck Institute for Infection Biology, Department of Immunology, Berlin, Germany; Archivel Farma; Fundació Institut d’Investigació en Ciències de la Salut Germans Trias i Pujol. Universitat Autònoma de Barcelona. CIBERES, Spain

## Abstract

Bacillus Calmette–Guérin (BCG), the only approved tuberculosis vaccine, provides only limited protection. Previously, we generated a recombinant derivative (BCG Δ*ureC*::*hly*), which secretes the pore-forming toxin listeriolysin O (LLO) of *Listeria monocytogenes*. This vaccine shows superior protection against tuberculosis in preclinical models and is safe in humans. Here we describe two new vaccine strains which express human interleukin-7 (hIL)-7 or hIL-18 in the genetic background of BCG Δ*ureC*::*hly* to modulate specific T cell immunity. Both strains exhibited an uncompromised *in vitro* growth pattern, while inducing a proinflammatory cytokine profile in human dendritic cells (DCs). Human DCs harbouring either strain efficiently promoted secretion of IL-2 by autologous T cells in a coculture system, suggesting superior immunogenicity. BALB/c mice vaccinated with BCG Δ*ureC*::*hly*, BCG Δ*ureC*::*hly_hIL7* or BCG Δ*ureC*::*hly*_*hIL18* developed a more robust Th1 response than after vaccination with parental BCG. Both strains provided significantly better protection than BCG in a murine *Mycobacterium tuberculosis* challenge model but efficacy remained comparable to that afforded by BCG Δ*ureC*::*hly*. We conclude that expression of hIL-7 or hIL-18 enhanced specific T cell responses but failed to improve protection over BCG Δ*ureC*::*hly* in mice.

## Introduction

An estimated 30% of the world’s population is latently infected with *Mycobacterium tuberculosis*, the aetiological agent of tuberculosis (TB) [1]. Of an estimated 8.7 million new TB cases worldwide in 2011, 1.4 million people died of whom 95% were from low- to middle-income countries [1]. In line with this, 1.1 million clinical TB cases have been reported to account for human immunodeficiency virus (HIV) coinfected individuals, with approximately 500,000 TB-related deaths globally [2], [3]. Additionally, the advent of drug-resistant *M. tuberculosis* strains complicates treatment while limiting chances of survival [4].

Vaccines remain the most cost-effective means to counteract the global challenges related to infectious diseases including TB [5]. Bacillus Calmette–Guérin (BCG) is the only licensed vaccine for TB, and protects children but leaves adults unprotected from the most prevalent form of the disease, pulmonary TB [6]. This calls for better vaccines against TB. Two main strategies are pursued in TB vaccine research – subunit and live vaccines. Subunit vaccines are generally aimed at boosting cellular immunity initially raised by BCG administered as prime vaccination [7]. Live vaccines are developed to replace BCG itself. Principally, a robust CD4^+^ T helper 1 (Th1) response represented by interferon gamma (IFN-γ) and tumour necrosis factor-alpha (TNF-α) expression should be induced by the prime vaccine to eventually form a pool of memory T cells which control TB disease [8], [9].

We have previously reported the superior protective efficacy of BCG Δ*ureC*::*hly* (VPM1002), a recombinant BCG vaccine candidate that is urease-deficient and heterologously expresses listeriolysin O (LLO) [10], [11]. One of the main features of this strain is its ability to perforate the host cell phagosomal membrane, thus releasing antigens into the cytosol of the host macrophage and promoting crosspriming [7], [10], [12].

Interleukin (IL)-7 and IL-18 have been implicated in immunity to *M. tuberculosis* infection [13], [14], [15], [16]. More specifically, IL-7 is involved in homeostatic regulation of T- and B-cell proliferation in humans and mice [17]. Administration of purified recombinant IL-7 has been shown to influence recall T cell responses to *M. tuberculosis* infection with and without prior BCG vaccination [15],[16]. IL-18, induces IFN-γ secretion jointly with IL-12 as well as expression of TNF-α [18]. Both IFN-γ and TNF-α are proinflammatory cytokines critical in shaping Th1-mediated immune responses in TB [19]. Mice lacking expression of IL-18 are susceptible to *M. tuberculosis* infection [13],[20]. Moreover, high levels of IL-18 were detected in sera of patients with advanced pulmonary TB [21].

Expression of recombinant cytokines by BCG has been shown to promote better immunogenicity [22],[23],[24]. We therefore hypothesized that incorporating the expression of human (h)IL-7 or IL-18 into BCG Δ*ureC*::*hly* could improve its immunogenicity. In this study, we describe two newly-derived candidate derivatives of BCG Δ*ureC*::*hly* coexpressing either IL-7 or IL-18, namely BCG Δ*ureC*::*hly_hIL7* and BCG Δ*ureC*::*hly_hIL18*, respectively. Both strains were evaluated for their intracellular fitness in primary human cells, and their immunomodulatory properties as well as protective efficacies against aerosol challenge with *M. tuberculosis*.

## Materials and Methods

### Ethics Statement

All experimental procedures involving mice were performed in accordance with requirements of, and approval by, the State Office for Health and Social Services (Landesamt für Gesundheit und Soziales), Berlin, Germany under permission number G0307/11. Mice were sacrificed by cervical dislocation, and all efforts were made to minimize suffering and pain.

### Bacterial Strains and Growth Conditions

BCG SSI 1331 (American Type Culture Collection, #35733), BCG Δ*ureC*::*hly* (VPM1002; [10],[11]) and *M. tuberculosis* H37Rv (American Type Culture Collection, #27294) were grown in Middlebook 7H9 borth (Becton Dickinson) supplemented with 0.2% w/v glycerol, 0.05% w/v Tween 80, 10% v/v albumin-dextrose-catalase supplement (Becton Dickinson) (7H9-ADC) or on Middlebrook 7H11 agar (Becton Dickinson) containing 10% v/v oleic acid-albumin-dextrose-catalase enrichment (Becton Dickinson) and 0.2% w/v glycerol. Mycobacterial cultures were grown to the mid-log phase in 1 L roller bottles (450 cm^2^) at 37°C and 2 rpm. For vaccine stock preparations, bacilli were collected by centrifugation [3200 rpm, room temperature (RT)], washed with phosphate-buffered saline (PBS) and stored at –80°C as PBS suspension with additional 10% glycerol. Prior to vaccination, vials were thawed, and cells harvested and resuspended in an appropriate volume of PBS. For CFU enumeration, serial dilutions were performed in phosphate-buffered saline containing 0.05% Tween 80 (PBST80) and plated on Middlebrook 7H11 agar. Plates were incubated at 37°C for 3–4 weeks prior to counting.

### Generation of BCG Δ*ureC*::*hly_hlL7* and BCG Δ*ureC*::*hly_hlL18*

Full length human IL-7 cDNA was amplified from pCM-SPORT6-hIL7 (American Type Culture collection, #10436529) using forward primer: 5′-TTATGCATGATTGTGATATTGAAGGTAAAG-3′ (*Nsi*I) and reverse primer: 5′-TTGGTACCCTCGAGTCAGTGTTCTTTAG-3′ (*Kpn*I). Full length human IL-18 cDNA was amplified from pENhIL-18 (Kind gift from Dr. Franck Biet, INRA) using forward primer: 5′-TTATGCATTACTTTGGCAAGCTTGAATCTAA-3′ (*Nsi*I) and reverse primer: 5′-ATGGTACCCTCGAGCTAGTCTTCGTTTTG-3′ (*Kpn* I). Secretion apparatus spanning the *groEL2* promoter and *fbpB* signal sequence was amplified from pAT261:Hly [25] using forward primer: 5′-TATCTAGACAAGGTCGAACGAGGGGCA-3′ (*Xba*I) and reverse primer: 5′-ATATGCATCGCGCCCGCGGTTG-3′ (*Nsi*I). All PCR amplifications were performed with the QIAGEN Taq Polymerase Kit (QIAGEN). pMVhIL-7 and pMVhIL-18 were generated by cutting amplicons with respective restriction enzymes and ligating *hIL-7* or *hIL-18* at the 3′end of the secretion apparatus (*Nsi*I overhang) using the Rapid DNA Ligation Kit (Roche) in the integrative vector pMV306 [26]. The identity of all constructs was confirmed by automated sequencing. BCG Δ*ureC*::*hly* has been previously described [10]. Briefly, this strain was generated by disrupting the *ureC* locus in the chromosome with DNA construct harbouring the *hly* gene under the control of the *groEL2* promoter fused to the *fbpB* secretion sequence, via a double-homologous recombination event marked by hygromycin resistance. The antibiotic marker was subsequently removed from the resulting strain as described previously [27]. Recombinant BCG Δ*ureC*::*hly* expressing *hIL-7* (BCG Δ*ureC*::*hly_hlL7*) or *hIL-18* (BCG Δ*ureC*::*hly_hlL18*) was generated by electroporation and selection of transformants on Middlebrook 7H11 agar containing 25 µg/mL kanamycin. New transformants were confirmed by PCR reactions using specific primers for *hIL-7* (5′-TGAAGGTAAAGATGGCAAACA-3′ and 5′-TCAGTGTTCTTTAGTG-3′) and *hIL-18* (5′-CTTTGGCAAGCTTGAAT-3′ and 5′-AGCTAGTCTTCGTTTGA-3′). The following primers were used to confirm transcription of *hly* (forward primer: 5′-ATTCATTAACACTCAGCATT-3′ and 5′-AGATATATGCAGGAGGATTT-3′) by reverse transcription PCR (RT-PCR).

### Infection of Primary Human Macrophages and Dendritic Cells

CD14^+^ primary human monocytes were purified from peripheral blood mononuclear cells (PBMCs, provided by Charité Hospital, Berlin, Germany) by magnetic separation on an LS column using antihuman CD14 MACS positive selection beads (Miltenyi). Cell pellets of purified CD14^+^ human monocytes were then resuspended in 20 mL of sterile RPMI medium (Gibco) supplemented with 10% fetal calf serum, 1% L-glutamine, 1% HEPES and 0.1% 2-mercaptoethanol (complete RPMI). After determining cell density, the suspension was equally divided in tissue culture flasks. Dendritic cells (DCs) were generated by addition of 50 ng/mL human GM-CSF and 25 ng/mL human IL-4, while macrophages (MΦs) were generated by addition of 50 ng/mL, human GM-CSF over 6 days of incubation at 37°C with 5% CO_2_. For infections, 10 mL of bacterial cultures grown to logarithmic phase (OD_600_ ∼0.6) were pelleted at 4000 rpm for 5 min at RT. Bacteria were washed with PBS and subsequently dislodged in 1 mL PBS. Single cell suspensions were prepared using a 23G needle (Braun, Germany) over 5 syringe-driven plunges. Human cells were infected at a multiplicity of infection (MOI) of 10 in 48-well culture plates and incubated at 37°C with 5% CO_2_ for 6 days.

### Coculture of Primary Human T Cells with Autologous-infected DCs

Primary human T cells were purified via magnetic separation on an LS column using the pan T cell isolation kit (Miltenyi). Purified T cells were coincubated with infected DCs at a ratio of 20 T cells per infected DC over a 24-h period at 37°C with 5% CO_2_. Cell-free culture supernatants were collected at designated time points by eliminating cellular debris using Costar Spin-X columns (Corning, USA). The flow-through was then stored at –20°C for multiplex or ELISA assays. Infected cells were washed once with PBS (1500 rpm, 5 min, RT) and lysed with 100 µL PBS/1% Triton X-100 for 10 min prior to plating in serial dilutions on Middlebrook 7H11 agar.

### ELISA, Multiplex Cytokine Assays and Flow Cytometry

The Bio-Rad Pro Human Cytokine 27-plex Panel was used for primary human MΦ and DC experiments while the Mouse Cytokine 23-plex Panel was used for analysis of cytokines in sera of vaccinated mice. In all multiplex assays, the volume of the coupled beads, detection antibodies and streptavidin-PE conjugate was halved and topped up with the appropriate buffer. The assays were otherwise performed according to the manufacturer’s instructions. Assay plates were read using the Bio-Plex 200 system (Bio-Rad). DuoSet human IL-7 and IL-2 ELISA kits were purchased from R&D Systems. Human IL-18 matched ELISA antibody pairs were purchased from eBioscience. Flow cytometric experiments were performed on a LSRII cytometer (BD Biosciences) and analyses were carried out using the FlowJo software (Tree Star Inc.). Antimouse antibodies for flow cytometry used in this study were as follows: antiCD3ε-Alexa Flour 700; clone: 17A2 (eBioscience), antiCD4-V500; clone RM4-5 (BD Horizon), antiCD8α-PerCP; clone: 169 (in-house), antiCD44-Pacific Blue; clone: IM7 (in-house), antiCD154-APC; clone: MR1 (eBioscience), antiIFN-γ-PE-Cy7; clone: XMG1.3 (BioLegend) and antiTNF-α-FITC; clone: XT-22 (in-house).

### Analysis of CD4^+^ T Cells from Vaccinated Mice

All *in vivo* experiments were carried out with 9- to 10-week old female BALB/c mice purchased from Janvier (France). Spleens, lungs and draining (inguinal) lymph nodes (dLNs) of mice were aseptically removed and individually homogenized in 2 mL of complete RPMI medium with penicillin and streptomycin (cRPMI+p/s) using 70-µm cell strainers (BD) and 2-mL syringe plungers to prepare single-cell suspensions (SCS). Cell strainers were washed with 12 mL of cRPMI +p/s, and along with the SCS centrifuged at 1300 rpm for 5 min at 4°C. Lungs were first cut into small pieces, digested with 10 mL of collagenase mix (0.7 mg/mL Collagenase IV (Sigma-Aldrich) and 0.3 mg/mL Collagenase D (Roche) in cRPMI+p/s) and incubated at 37°C with 5% CO_2_ for 30 min prior SSC preparation. Erythrocytes in samples were lysed by addition of 2 mL 0.0083% NH_4_Cl/0.001% KHCO_3_/0.037% EDTA/H_2_O at RT for 2 min. Subsequently lysis was stopped by 12 mL PBS/0.2% bovine serum albumin and cells were pelleted at 1300 rpm for 5 min at RT. The dLN and lung cell pellets were resuspended in 500 µL cRMPI+p/s and spleen cell pellets in 3 mL cRPMI+p/s. Five µL of the respective suspensions was added to 200 µL of 1x AccuCheck bead suspension (Invitrogen; 1∶5 diluted) for flow cytometric counting. A total of 500 µL of each cell suspension was seeded in a 48-well plate, and stimulated with 10 µg/mL of *M. tuberculosis* H37Rv whole cell lysate in presence of 5 µg/mL brefeldin A for 6 h at 37°C/5% CO_2_. Cells were then washed, resuspended in 200 µL of PBS-BSA and transferred to a 96-well microtitre filter plate. After centrifugation (1300 rpm, 3 min) cell pellets were resuspended in 90 µL PBS-BSA and stained on ice for 20 min with 10 µL of a 10× surface stain master mix containing antiCD4-V500, antiCD44-Pacific Blue, antiCD3-Alexa Fluor 700 and antiCD8-PerCP at 1∶20 dilution. Afterwards, cells were washed with 200 µL of PBS-BSA, centrifuged (1300 rpm, 3 min), resuspended in 60 µL of BD Cytofix/Cytoperm solution and incubated on ice for 20 min. Following one wash step with 150 µL perm wash (BD), cell pellets were resuspended in 50 µL of the following antibodies in perm wash for intracellular staining on ice for 30 min (antiCD154-APC; 1∶20 dilution), antiTNF-α-FITC and antiIFN-γ-PECy7, 1∶200 dilution). This was followed by a final wash step with 150 µL perm wash and resuspended in 200 µL PBS-BSA. The stained cell suspension was applied to a 35 µm cell strainer capped onto a 12×75 mm tube (BD) for a quick spin followed by flow cytometry analysis.

### Safety and Protective Efficacy of rBCG Strains in Mice

Mice were vaccinated subcutaneously in the tail base with 10^6^ CFUs of either BCG SSI 1331, BCG Δ*ureC::hly*, BCG Δ*ureC::hly_hlL7* or BCG Δ*ureC::hly_hIL18*. At designated time points post-vaccination, spleens and dLNs of vaccinated mice were aseptically removed for CFU enumeration. For the protective efficacy study, mice were challenged via the aerosol route with 200 CFUs of *M. tuberculosis* H37Rv 90 days post-vaccination. At designated time points, lungs and spleens were aseptically removed, homogenized in PBS/0.05% Tween 80 and plated in serial dilutions onto 7H11 agar for CFU enumeration.

### Statistical Analyses

The GraphPad Prism 6 software was used for data analysis. Results were expressed as mean ± standard error of the mean (SEM) and analyzed using one-way or two-way analysis of variance (ANOVA) and Bonferroni’s or Tukey’s multiple comparisons post-test as applicable. Value of *p<0.05* was considered statistically significant.

## Results

### Generation of BCG Δ*ureC*::*hly_hIL7* and BCG Δ*ureC*::*hly_hIL18*

**Figure 1:**
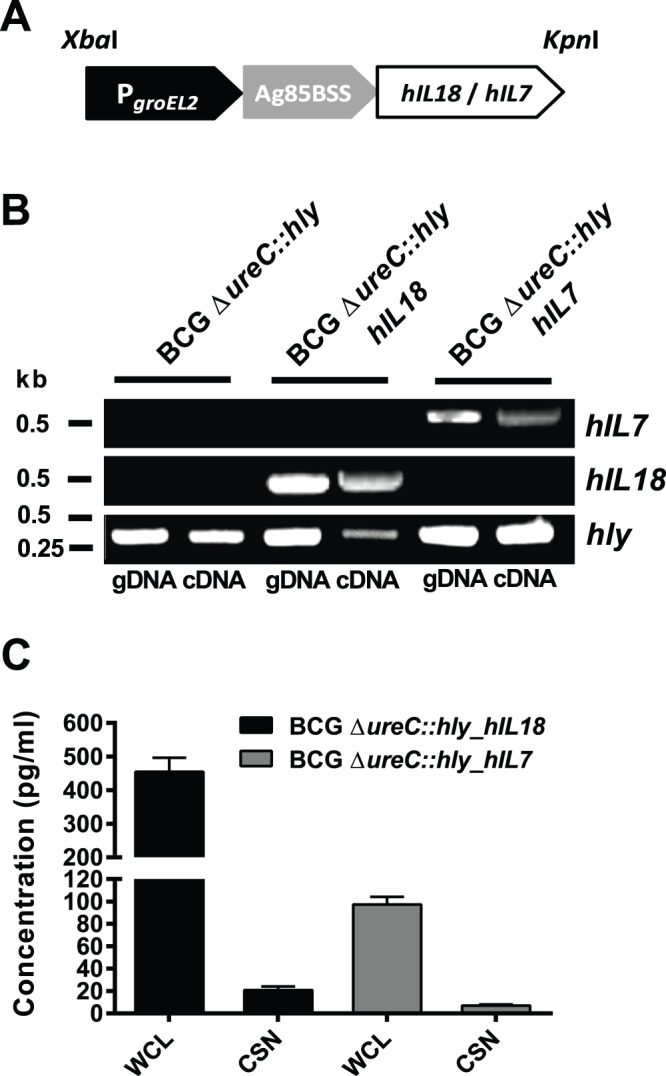
Generation and validation of BCG Δ*ureC*::*hly*_*hIL7* and BCG Δ*ureC*::*hly*_*hIL18*. **A**. Organisation of the cytokine expression cassette for stable transformation of BCG Δ*ureC*::*hly*. **B**. Confirmation of hIL-7 and hIL-18 gene presence (gDNA as template) and transcription (cDNA as template) in BCG Δ*ureC:hly_hIL7* or BCG Δ*ureC:hly_hIL18*, respectively using PCR or RT-PCR. **C**. Confirmation of hIL-18 and hIL-7 protein expression by the respective strains using ELISA. Experiments were performed twice with similar outcome.

We first confirmed presence of the hIL-7 or the hIL-18 gene in the respective BCG Δ*ureC*::*hly* transformants by PCR using genomic DNA (gDNA) as template and specific oligonucleotides. A schematic representation of the IL expression cassette is shown in Figure 1A. Cytokine gene and *hly* transcription was then investigated by RT-PCR. Expression of LLO is an integral feature of the parental strain, BCG Δ*ureC*::*hly* and its derivatives. The mRNA level of *hIL-7* was lower than that of *hIL-18* produced by the respective strain (Figure 1B). However, the stronger transcription of *hIL-18* was associated with reduced amounts of *hly* mRNA which encode LLO, the integral component of BCG Δ*ureC*::*hly*. ELISA analysis of whole cell extracts and culture supernatants revealed that our observation with gene transcription of hIL-7 and hIL-18 correlated with hIL-7 and hIL-18 protein expression by BCG Δ*ureC*::*hly_hIL7* and BCG Δ*ureC*::*hly_hIL18*, respectively (Figure 1C). However, the majority of protein was accumulated in the cytosolic fraction and only minor quantities were secreted.

### BCG Δ*ureC*::*hly_hIL18* Exhibits a Proinflammatory Phenotype *in vitro*

**Figure 2:**
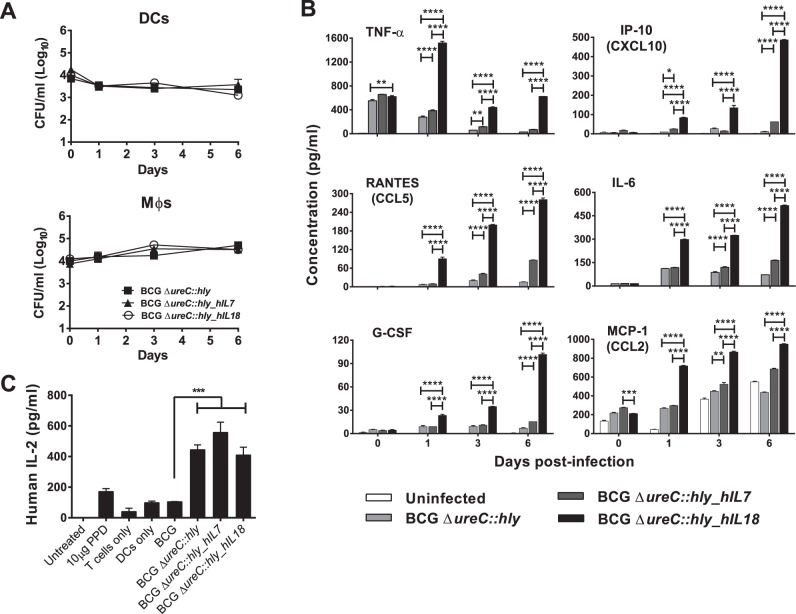
BCG Δ*ureC*::*hly_hIL18* exhibits a proinflammatory phenotype *in vitro*. **A**. Intracellular growth dynamics of BCG Δ*ureC*::*hly*_*hIL7* and BCG Δ*ureC*::*hly*_*hIL18* in primary human dendritic cells (DCs, top panel) and macrophages (MΦ, bottom panel). **B**. Cytokine secretion profile of DCs infected with BCG Δ*ureC*::*hly*, BCG Δ*ureC*::*hly*_*hIL7* or BCG Δ*ureC*::*hly*_*hIL18*. Shown are means ± SEM of duplicate cultures analyzed using two-way ANOVA and Tukey’s post-hoc test; *p<0.05; **p<0.01; ***p<0.001; ****p<0.0001. **C**. Secretion of IL-2 by activated human T cells following coculture with rBCG-infected autologous DCs. Shown are means ± SEM analyzed using one-way ANOVA and Tukey’s post-hoc test; ***p<0.001. Experiments were performed twice.

The intracellular growth and survival kinetics of BCG Δ*ureC*::*hly_hIL7* and BCG Δ*ureC*::*hly_hIL18* were studied in primary human MΦs and DCs. BCG Δ*ureC*::*hly* served as control in these experiments. The growth kinetics of BCG Δ*ureC*::*hly*, BCG Δ*ureC*::*hly_hIL7* and BCG Δ*ureC*::*hly_hIL18* were similar in both MΦs and DCs, indicating that recombinant cytokine expression did not compromise intracellular fitness of the strains under investigation (Figure 2A). Intriguingly, bacterial growth was more efficiently restricted in DCs than in MΦ. Secretion of a panel of proinflammatory cytokines, including IL-6, TNF-α and G-CSF was highly upregulated in DCs infected with BCG Δ*ureC*::*hly_hIL18* (Fig. 2B), indicating that the potential of BCG Δ*ureC*::*hly_hIL18* to initiate proinflammatory cytokine signalling promoted Th1 responses. However, we did not observe such differences with infected MΦs (data not shown).

In order to investigate whether BCG Δ*ureC*::*hly_hIL7* and BCG Δ*ureC*::*hly_hIL18* enhance T cell activation, we infected primary human DCs with either strain and cocultured them with autologous T cells over a 24-h period. The secretion of IL-2, a cytokine important for T cell proliferation, was chosen as readout. BCG Δ*ureC*::*hly*, BCG Δ*ureC*::*hly_hIL7* and BCG Δ*ureC*::*hly_hIL18* were all significantly better at priming T cells compared to BCG as determined by IL-2 secretion (Figure 2C). Taken together, expression of IL-7 or IL-18 in the genetic background of BCG Δ*ureC::hly* did not influence T cell activation in this model.

### BCG Δ*ureC*::*hly_hIL18* Induces a Proinflammatory Cytokine Response in Mice

**Figure 3:**
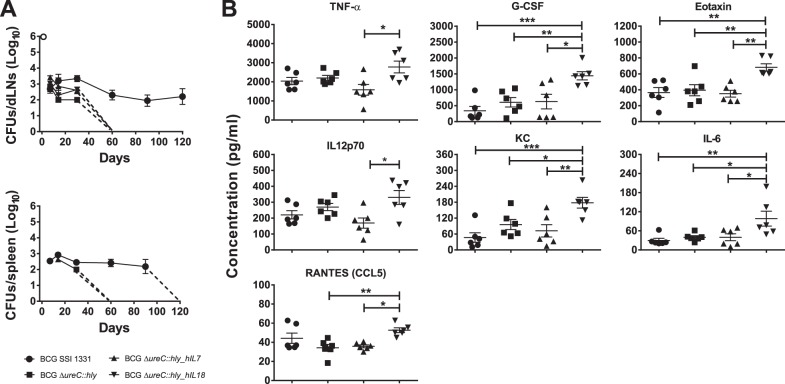
*In vivo* characterisation of BCG Δ*ureC*::*hly*_*hIL7* and BCG Δ*ureC*::*hly*_*hIL18*. **A**. Dissemination and clearance of BCG Δ*ureC*::*hly*_*hIL7* and BCG Δ*ureC*::*hly*_*hIL18* from draining (inguinal) lymph nodes (dLNs) and spleens of vaccinated mice. The detection limit for enumeration of colony forming units (CFUs) was 20 per organ. Shown are means ± SEM (n = 3 mice per group). **B**. Serum cytokine responses in vaccinated mice. Significant differences were observed only on day 7 post-vaccination. Shown are means ± SEM (n = 6 mice per group) analyzed using one-way ANOVA and Tukey’s post-hoc test; *p<0.05; **p<0.01 ***p<0.001. Experiments were performed twice.

One of the desired features of a live TB vaccine is prompt clearance from the host as a measure of safety. We vaccinated mice with BCG SSI 1331, BCG Δ*ureC*::*hly*, BCG Δ*ureC*::*hly_hIL7* or BCG Δ*ureC*::*hly_hIL18* and monitored dissemination to as well as clearance from the spleen and draining (inguinal) lymph nodes (dLNs). All rBCG strains were cleared within 60 days post-vaccination from both organs while BCG SSI 1331 was found in the dLNs up to 120 days post-vaccination (Figure 3A). However, no bacteria were observed in the spleen beyond 90 days post-vaccination. Thus, improved clearance and indirectly safety of BCG Δ*ureC*::*hly* was preserved in both derivative strains under investigation.

We investigated serum cytokine levels using a multiplex platform for 27 cytokines. All comparisons were made in relation to BCG Δ*ureC*::*hly*. In contrast to the derivative strain expressing hIL-7, BCG Δ*ureC*::*hly_hIL18* vaccinated mice showed up-regulation of proinflammatory cytokines IL-6, KC, CCL5, IL-2 and G-CSF (Figure 3B). In more detail, KC is a murine orthologue of human IL-8, an important neutrophil chemoattractant [28]. In line with this, G-CSF is instrumental in proliferation of neutrophils [29]. IL-6 is involved in T cell proliferation and B cell stimulation [30],[31] as well as prostaglandin release [32]. IL-2 also reflects T cell proliferation while CCL5 plays a role in T cell chemotaxis [33],[34]. This observation was consistent with the cytokine analysis performed with culture supernatants of infected human DCs, confirming the proinflammatory potential of BCG Δ*ureC*::*hly_hIL18* as compared to its parental strain (Figure 2C).

### BCG Δ*ureC*::*hly_hIL7* and BCG Δ*ureC*::*hly_hIL18* enhance antigen-specific Th1 responses

The main objective of this study was to assess the effect of human cytokine coexpression by BCG Δ*ureC*::*hly* on T cell responses. Mice vaccinated with BCG SSI 1331, BCG Δ*ureC*::*hly*, BCG Δ*ureC*::*hly_hIL7* or BCG Δ*ureC*::*hly_hIL18* were monitored for modulation of *M. tuberculosis*-specific CD4^+^ T cells based on their expression of CD40L over time. *M. tuberculosis* H37Rv whole cell lysate was used as source of T cell antigens.

**Figure 4:**
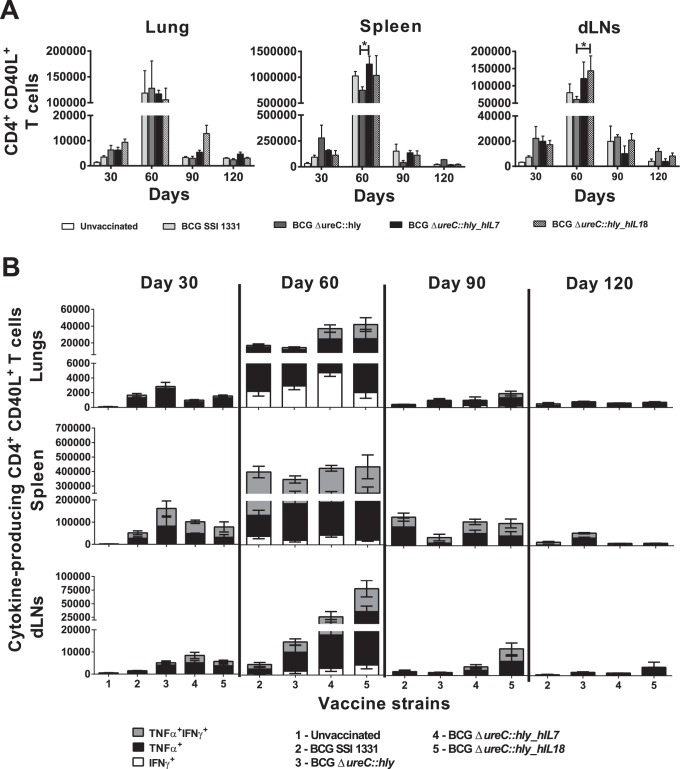
BCG Δ*ureC*::*hly_hIL7* and BCG Δ*ureC*::*hly_hIL18* enhance antigen-specific Th1 responses. Flow cytometric analysis of CD40L-expressing antigen-specific CD4^+^ T cell responses (**A**) and cytokine-producing CD40L^+^CD4^+^ T cells (**B**) in the lungs, spleen and draining (inguinal) lymph nodes (dLNs) of vaccinated mice following *in vitro* re-stimulation with *M. tuberculosis* H37Rv whole cell lysate. Only comparisons made with BCG Δ*ureC*::*hly* are displayed. Shown are means ± SEM (n = 3 mice per group) analyzed using two-way ANOVA and Tukey’s post-hoc test; *p<0.05. Experiment was performed twice.

On day 30 post-vaccination, lungs, spleens and dLNs of rBCG-vaccinated mice harboured similar numbers of activated CD4^+^ T cells (Figure 4A). At 60 days post-vaccination, BCG SSI 1331, BCG Δ*ureC*::*hly*, BCG Δ*ureC*::*hly_hIL7* and BCG Δ*ureC*::*hly_hIL18* elicited equal numbers of CD40L-expressing CD4^+^ T cells in lungs of vaccinated mice. However, BCG Δ*ureC*::*hly_hIL7* elicited significantly higher CD40L^+^CD4^+^ T cells than BCG Δ*ureC*::*hly* in the spleen (*p<0.05*), while BCG Δ*ureC*::*hly_hIL18* achieved a similar feat in the dLNs (*p<0.05*). Increased numbers of CD40L^+^CD4^+^ T cells in spleens between days 90 and 120 post-vaccination were only observed in mice which received BCG Δ*ureC*::*hly*.

In addition to CD40L^+^ expression, functionality of the *M. tuberculosis*-specific CD4^+^ T cells was also examined. BCG Δ*ureC*::*hly_hIL18* contributed to the largest proportions of CD40L^+^TNF-α^+^ and CD40L^+^TNF-α^+^IFN-γ^+^ CD4^+^ T cells on days 60 and 90 post-vaccination, respectively (Fig. 4B, Table 1). CD40L^+^IFN-γ^+^ CD4^+^ T cells were also elicited by the vaccine strains at day 60 post-vaccination, with the largest proportion in the lungs of BCG Δ*ureC*::*hly_hIL7*-vaccinated mice. In the dLNs, BCG Δ*ureC*::*hly_hIL18* and BCG Δ*ureC*::*hly_hIL7* induced an increase in CD40L^+^TNF-α^+^ and CD40L^+^TNF-α^+^IFN-γ^+^ CD4^+^ T cell numbers compared to BCG SSI 1331 or BCG Δ*ureC*::*hly* between 30 and 60 days post-vaccination. A similar observation was made in the lungs and spleen at 60 days post-vaccination. No CD40L^+^IFN-γ^+^ CD4^+^ T cells were seen in any of the vaccinated mice after day 60 post-vaccination. We conclude that vaccination of mice with either BCG Δ*ureC*::*hly_hIL7* or BCG Δ*ureC*::*hly_hIL18* induced specific CD4^+^ T cell responses lasting up to at least 90 days. Particularly, BCG Δ*ureC*::*hly_hIL18* was capable of inducing a proinflammatory immune response early on, which triggers enhanced CD4^+^ T cell responses up to 90 days post-vaccination.

**Table 1:** Evaluation of cytokine expression by antigen-specific CD4^+^ T cells.

Time pointpost vaccination	Organ	Vaccinated groups compared	Cytokine(s) secreted byantigen-specific CD4+ T cells	Statisticalsignificance^†^
D30	Lung	Naïve vs. BCG Δ*ureC*::*hly*	TNF-α	****
		BCG Δ*ureC*::*hly* vs. BCG Δ*ureC*::*hly_hIL7*	TNF-α	***
		BCG Δ*ureC*::*hly* vs. BCG Δ*ureC*::*hly_hIL18*	TNF-α	*
	Spleen	Naïve vs. BCG Δ*ureC*::*hly*	TNF-α	*
			TNF-α, IFN-γ	*
	LN	Naïve vs. BCG Δ*ureC*::*hly_hIL7*	TNF-α	**
D60	Lung	No difference		
	Spleen	No difference		
	LN	BCG SSI 1331 vs. BCG Δ*ureC*::*hly_hIL18*	TNF-α	*
			TNF-α, IFN-γ	**
		BCG Δ*ureC*::*hly* vs. BCG Δ*ureC*::*hly_hIL18*	TNF-α, IFN-γ	*
D90	Lung	No difference		
	Spleen	No difference		
	LN	No difference		
D120	Lung	No difference		
	Spleen	BCG SSI 1331 vs. BCG Δ*ureC*::*hly*	TNF-α	****
		BCG Δ*ureC*::*hly* vs. BCG Δ*ureC*::*hly_hIL7*	TNF-α	****
			TNF-α, IFN-γ	***
		BCG Δ*ureC*::*hly* vs. BCG Δ*ureC*::*hly_hIL18*	TNF-α	****
			TNF-α, IFN-γ	****
	LN	BCG SSI 1331 vs. BCG Δ*ureC*::*hly_hIL18*	TNF-α	*

Two-way ANOVA and Bonferroni multiple comparisons test were performed to evaluate the statistical significance between the expression of TNF-α, IFN-γ or both by antigen-specific CD4^+^ T cells derived from lungs, spleen or draining (inguinal) lymph nodes (LNs) of vaccinated mice following *in vitro* restimulation with *M. tuberculosis* H37Rv whole cell lysate. Shown are means ± SEM of 3 mice per group. †, *p<0.05; **p<0.01; ***p<0.001; ****p<0.0001.

### Protective Efficacy of BCG Δ*ureC*::*hly*_*hIL7* and BCG Δ*ureC*::*hly*_*hIL18*

**Figure 5:**
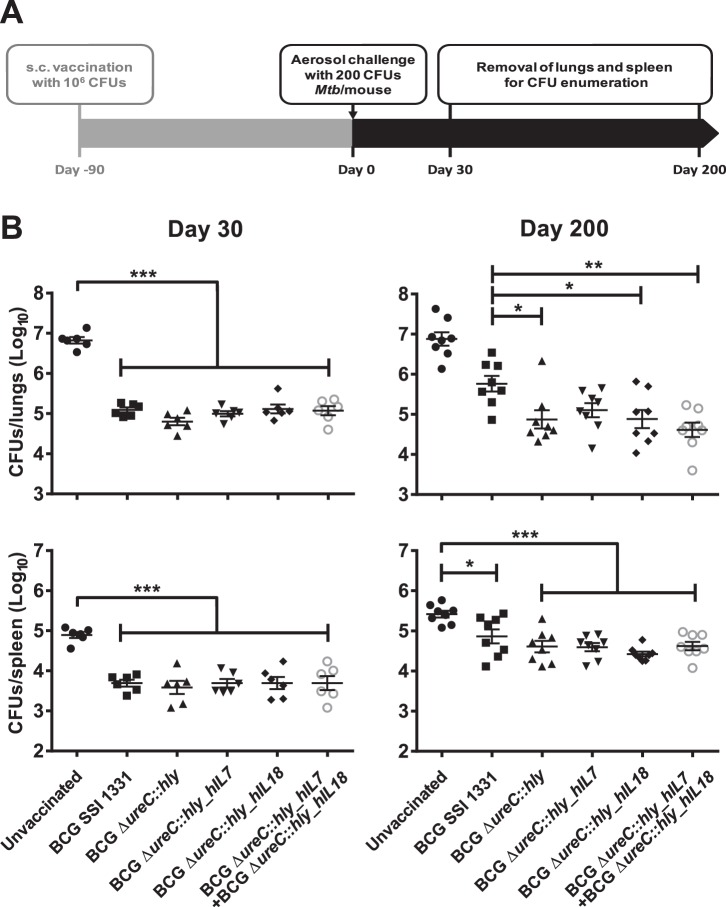
BCG Δ*ureC*::*hly*_*hIL7* and BCG Δ*ureC*::*hly*_*hIL18* emulate the protective efficacy of BCG Δ*ureC*::*hly*. **A**. Experimental design for protection study. **B**. Protective efficacy of BCG Δ*ureC*::*hly*_*hIL7* and BCG Δ*ureC*::*hly*_*hIL18* in a murine aerosol challenge model of *M. tuberculosis*. Shown are means ± SEM (n = 6–8 mice per group) analyzed using one-way ANOVA and Tukey’s post-hoc test; *p<0.05; **p<0.01 ***p<0.001. Experiment was performed twice.

For validating the protective capacities of BCG Δ*ureC*::*hly_hIL7* and BCG Δ*ureC*::*hly_hIL18* against TB, vaccinated mice were challenged with *M. tuberculosis* H37Rv via the aerosol route and bacterial burdens in lungs and spleens were monitored over time. A schematic representation of the efficacy study is displayed in Figure 5A. In a vaccination group that received a mix of BCG Δ*ureC*::*hly_hIL7* and BCG Δ*ureC*::*hly_hIL18*, we aimed to analyze potential synergies. At 30 days post-aerosol challenge, mice vaccinated with BCG Δ*ureC*::*hly*, BCG Δ*ureC*::*hly_hIL7* or BCG Δ*ureC*::*hly_hIL18* showed similar protection as BCG SSI 1331 in lungs, while at day 200 all rBCG strains provided superior protection over BCG SSI, as reported earlier for BCG Δ*ureC*::*hly* (Figure 5B) [10]. In spleens, no differences between BCG strains were observed, but all strains conferred significant protection as compared to an unvaccinated control group. We conclude that expression of hIL-7 or hIL-18 in the genetic background of BCG Δ*ureC*::*hly* did not alter protective efficacy in mice.

## Discussion

BCG remains the most widely applied human vaccine in current use, with over 4 billion doses administered [6],[7]. Over the years, BCG has been cultured and passaged by various laboratories across the globe, giving rise to a range of genetically different BCG substrains [35]. BCG protects against severe forms of TB in children but not against the most prevalent pulmonary form in all age groups [6],[36]. Moreover, BCG is only to be given pre-exposure and therefore does not apply to individuals with latent TB infection (LTBI), who do not present with clinical symptoms but nonetheless are at 5–10% risk of developing clinical TB disease during their lifetime [37]. Various efforts are currently underway to improve antitubercular immunity by rBCG vaccines [38].

BCG was first considered as a vehicle for heterologous expression of foreign proteins with the development of mycobacterial expression vectors in the early 1990s [26]. Since then, BCG was used to express proteins from an array of human pathogens such as *Plasmodium falciparum* [39], the human immunodeficiency virus (HIV) [40], measles virus [41] *Streptococcus pneumonia* [42] and *Borrelia burgdoferi* [43], as well as immunomodulatory molecules such as cytokines, i.e. GM-CSF, IL-18, IFN-γ [22],[24] and others. Taken together, BCG proved to be a useful platform for heterologous antigen expression.

In this study, we designed and constructed experimental rBCG vaccine candidates coexpressing either human IL-7 or IL-18 in the genetic background of BCG Δ*ureC*::*hly*, a TB vaccine candidate that is in phase IIa clinical development [11] (ClinicalTrial.gov, #NCT01479972). The BCG Δ*ureC*::*hly* strain shows superior protection against TB in preclinical models as compared to canonical BCG [10]. The scientific rationale behind generation of this strain was to increase access of mycobacteria–derived antigens to the host’s antigen processing and presentation machinery to ultimately improve protective efficacy against TB. We hypothesized that by capitalising on the characteristics of BCG Δ*ureC*::*hly*, better potentiation of cellular immunity could be achieved using soluble mediators such as IL-7 or IL-18.

We expected that perforation of the phagosome by means of the thiol-activated perforin LLO containing either BCG Δ*ureC*::*hly_hIL7* or BCG Δ*ureC*::*hly_hIL18* could allow release of the recombinant cytokines into the cytosol of host cells thereby modulating T cell responses. IL-18, a highly inflammatory cytokine that is produced by macrophages probably plays a major role in protective immunity to TB [13],[20]. IL-7 primarily acts on T cells, notably resting memory T cells which express the IL-7 receptor alpha chain (IL-7Rα) [44]. Biological activity of human IL-7 and IL-18 was demonstrated in mice suggesting reasonable cross activity in both species [45].

Semi-quantitative RT-PCR of BCG Δ*ureC*::*hly_hIL7* and BCG Δ*ureC*::*hly_hIL18* using specific oligonucleotides revealed that gene transcription of *hIL-7* by the former was lower than *hIL-18* gene transcription by the latter (Figure 1B). Conversely, the level of *hly* transcripts in BCG Δ*ureC*::*hly_hIL18* was lower than that observed in all other rBCG strains (Figure 1B). Although *hly* and *hIL-18* are both controlled by the constitutive *groEL2* promoter (Figure 1A), transcription of *hIL-18* was favoured over *hly*. In contrast, the *groEL2* promoter-driven *hly* transcription remained uncompromised in BCG Δ*ureC*::*hly_hIL7* while *hIL-7* transcript levels were reduced (Figure 1B). Consistent with this, abundance of hIL-7 protein expressed by BCG Δ*ureC*::*hly_hIL7* was lower than that of hIL-18 expressed by BCG Δ*ureC*::*hly_hIL18* (Figure 1C). The fact that cytokine genes were not translated into GC-rich mycobacterial codon usage [46] prior introduction into the vaccine strain might have affected expression of hIL7 and hIL18.

BCG Δ*ureC*::*hly_hIL18* seemed to induce more profound proinflammatory cytokine release compared to BCG Δ*ureC*::*hly* in human DCs although the number of intracellular bacteria did not significantly differ between the strains (Figures 2A and B). This may be due to the physiological effects of vaccine-derived human IL-18 present in the culture on human DCs. Up-regulation of chemokines such as CXCL10, CCL2 and CCL5 indicates that BCG Δ*ureC*::*hly_hIL18* could enhance recruitment of monocytes and T cells to the site of vaccination, thus amplifying early immunological events in shaping T cell responses [47],[48],[49],[50]. G-CSF contributes to development of neutrophils, which have been implicated in shuttling BCG to dLNs for T cell priming [51]. Collectively, these features could facilitate generation of long-lived mycobacterial antigen-specific T cells shortly after vaccination. We also observed enhanced IL-2 secretion triggered by all three rBCG strains, indicating T cell activation and proliferation (Figure 2C). We did not expect to observe similar cytokine profiles in culture supernatants of DCs infected with BCG Δ*ureC*::*hly_hIL7* since IL-7 is not involved in proinflammatory immune responses but rather in T cell homeostasis [52].

The improved clearance patterns of all our recombinant vaccines from mice suggests a better safety profile compared to BCG (Figure 3A), in line with the requirements of a live TB vaccine candidate aiming to replace BCG, especially since BCG can cause disseminated disease termed BCGosis in HIV-positive patients [53]. Urease deletion in mycobacteria has been shown to prevent microbe-driven deacidification of the phagosome in antigen-presenting cells (APCs) [54]. Since this feature complements the optimal biological activity of LLO at pH 5.5, BCG Δ*ureC*::*hly* facilitates higher cytosolic antigen turnover and thus, enhanced apoptosis of the APCs, which in turn allows for efficient cell-mediated immune attack and clearance of the BCG Δ*ureC*::*hly* strains [10]. Moreover, we found higher levels of proinflammatory cytokines such as CCL5, G-CSF and IL-6 (among others) in sera of BCG Δ*ureC*::*hly_hIL18*-vaccinated mice compared to those vaccinated with BCG Δ*ureC*::*hly*, confirming the cytokine profile we observed with infected human DCs (Figure 3B).

Protective immunity to TB is largely orchestrated by antigen-specific Th1 CD4^+^ T cells [5]. Upon establishing a successful immune synapse with an epitope-loaded major histocompatibility complex class II (MHC-II) molecule on the surface of an antigen presenting cell (APC), CD4^+^ T cells stably express the activation marker CD40L (or CD154), thereby indicating antigen-specific stimulation [55]. *In vitro* re-stimulation with *M. tuberculosis* H37Rv whole cell lysate revealed that mice vaccinated with BCG Δ*ureC*::*hly_hIL7* or BCG Δ*ureC*::*hly_hIL18* responded to specific antigenic challenge (measured by CD40L expression) by a magnitude of 10-fold compared to unvaccinated mice (Figure 4A). In addition, the modulation of IFN-γ- and TNF-α-producing Th1 CD4^+^ T cells in dLNs, spleens and lungs of BCG Δ*ureC*::*hly_hIL7-* or BCG Δ*ureC*::*hly_hIL18-*vaccinated mice hint to favourable immunity elicited by both strains (Figure 4B). Yet, the protective efficacy afforded by BCG Δ*ureC*::*hly_hIL7* and BCG Δ*ureC*::*hly_hIL18* was not superior to that of BCG Δ*ureC*::*hly* in the mouse model (Figure 5B).

Analysis of BCG Δ*ureC*::*hly_hIL7* and BCG Δ*ureC::hly_hIL18* in non-murine model systems may be considered in the future. Other reasons for the lack of improved efficacy of cytokine-expressing vaccine candidates might be: (i) Low abundance of hIL-7 and hIL-18 as seen in the culture supernatant analysis in Figure 1C. Stronger secretion capacity may have resulted in higher cytokine content in the host MΦ cytosol and hence, enhanced cytokine export via the endogenous secretion system. (ii) Overload of the mycobacterial protein export machinery due to use of the same export system (P*_groEL2_*-Ag85BSS) both for LLO and cytokines. This was already reflected in gene transcription efficiency (semi-quantitative RT-PCR data; Figure 1B) as a direct effect of compromised *groEL2* promoter activity.

Taken together, our data suggest that expression of human IL-7 or IL-18 in BCG allows modulation of immune reactions in response to vaccination. More work is required in order to elucidate mechanisms which contribute to protective immunity against *M. tuberculosis*. Efforts are currently underway in our laboratory to better understand the molecular basis of immunomodulation driven by BCG Δ*ureC*::*hly*. This may lead to development of alternative strategies to improve the already potent protection elicited by BCG Δ*ureC*::*hly*.
